# Simulating and Comparing CO_2_/CH_4_ Separation Performance of Membrane–Zeolite Contactors by Cascade Neural Networks

**DOI:** 10.3390/membranes13050526

**Published:** 2023-05-18

**Authors:** Seyyed Amirreza Abdollahi, AmirReza Andarkhor, Afham Pourahmad, Ali Hosin Alibak, Falah Alobaid, Babak Aghel

**Affiliations:** 1 Faculty of Mechanical Engineering, University of Tabriz, Tabriz 5166616471, Iran; 2 Department of Chemistry, Payam Noor University (Bushehr Branch), Bushehr 1688, Iran; 3 Department of Polymer Engineering, Amirkabir University of Technology, Tehran 1591634311, Iran; 4 Chemical Engineering Department, Faculty of Engineering, Soran University, Soran 44008, Iraq; 5 Faculty of Chemical and Petroleum Engineering, University of Tabriz, Tabriz 5166616471, Iran; 6 Institut Energiesysteme und Energietechnik, Technische Universität Darmstadt, Otto-Berndt-Straße 2, 64287 Darmstadt, Germany; 7 Department of Chemical Engineering, Faculty of Energy, Kermanshah University of Technology, Kermanshah 6715685420, Iran

**Keywords:** CO_2_/CH_4_ gas mixture, membrane separation, selectivity, intelligent modeling

## Abstract

Separating carbon dioxide (CO_2_) from gaseous streams released into the atmosphere is becoming critical due to its greenhouse effect. Membrane technology is one of the promising technologies for CO_2_ capture. SAPO-34 filler was incorporated in polymeric media to synthesize mixed matrix membrane (MMM) and enhance the CO_2_ separation performance of this process. Despite relatively extensive experimental studies, there are limited studies that cover the modeling aspects of CO_2_ capture by MMMs. This research applies a special type of machine learning modeling scenario, namely, cascade neural networks (CNN), to simulate as well as compare the CO_2_/CH_4_ selectivity of a wide range of MMMs containing SAPO-34 zeolite. A combination of trial-and-error analysis and statistical accuracy monitoring has been applied to fine-tune the CNN topology. It was found that the CNN with a 4-11-1 topology has the highest accuracy for the modeling of the considered task. The designed CNN model is able to precisely predict the CO_2_/CH_4_ selectivity of seven different MMMs in a broad range of filler concentrations, pressures, and temperatures. The model predicts 118 actual measurements of CO_2_/CH_4_ selectivity with an outstanding accuracy (i.e., AARD = 2.92%, MSE = 1.55, R = 0.9964).

## 1. Introduction

Developing carbon capture and sequestration technologies, deploying renewable sources of energy, and tightening the regulations are the key strategies to achieve the Paris Agreement targets [1,2]. To this end, among different renewable energies such as solar, wind, biogas, and biomass, biogas has already demonstrated an appealing potential to be replaced with fossil fuels [3]. On the other hand, to meet the pipeline safety and maintenance criteria, biogas requires upgrading by separating the bio-methane from the involved contaminants, such as carbon dioxide [4]. Accordingly, separating the CO_2_/CH_4_ is not only necessary for climate change mitigation, but also favorable for synthesizing the value-added chemicals [5]. To date, several different technologies including electrochemical reduction [6], membrane [7], cryogenic [8], adsorption [9], and absorption [10] have been investigated for gas capture and sequestration. Selectivity is one of the key factors of the membrane-based separation processes [11].

Among these strategies, absorption and cryogenic ones are the most mature, while absorption represents some serious environmental problems and cryogenics consume a high amount of energy [12,13]. In addition, membrane technology which is efficient and environmentally friendly appears as the most interesting for gas capture and separation [14]. On these grounds, in recent decades, numerous studies have been devoted to developing different types of membranes or improving their performance [14]. Some of the recent interesting ones can be found in [14,15].

Routinely, polymeric membranes are known for their high modular specific area, and ease of processing [16,17], while the low separation performance because of the Robeson upper bound is the major drawback of these types of membranes [18,19]. Furthermore, low permeability and selectivity are the other limitations of polymeric membranes for large-scale sequestration [20]. To cover these impediments, mixed matrix membranes (MMMs) emerged to improve the polymer’s separation performances by incorporating the textural characters of carbon molecular sieves or zeolites in the conventional polymer matrixes [19]. It is worth mentioning that adding the inorganic fillers to the polymeric matrix and developing highly efficient membranes involves some hardness, including weak contact and/or poor distribution of the fillers in the considered polymer matrix [14,19]. Furthermore, fabricating MMMs is influenced by the loading of filler, particle size, polymer properties, and also filler pore size [14,21].

Accordingly, zeolites based on their particular characteristics, i.e., crystallinity, uniformity, and ion exchange potential, have provided an excellent capacity for different areas of gas capture and sequestration [22,23]. Among various introduced zeolites, SAPO-34 zeolite is considered a unique one concerning its interesting specifications including pore diameter, medium acidity, selectivity, high stability, and kinetic diameter for separating CH_4_ and CO_2_ in related applications such as biogas upgrading and natural gas sweetening [24,25]. Many researchers experimentally investigated the CO_2_/CH_4_ selectivity of the MMMs containing SAPO-34 as a function of pressure, temperature, filler dosage, and polymer type (see the next section). The review article written by Rimaz et al. presents several interesting illustrations of the SAPO-34 zeolite which help readers understand the chemistry of this zeolite [26]. Routinely, selectivity experimental measurement is costly, time-consuming, and is affected by human-caused errors.

On the other hand, machine learning (ML) approaches have received significant interest for the intelligent simulating of a wide range of problems [27,28,29]. To this end, fault detection [30], pattern recognition [31], data mining [32], and model deriving [33] are some of the main covered domains by ML. Lately, several different ML topologies, including artificial neural networks (ANN) [34], support vector machines [35], adaptive neuro-fuzzy inference systems (ANFIS) [36], and genetic programming [37] have been employed in membrane technology. In this way, Zhao et al. evaluated membrane-fouling criteria in a membrane bioreactor for the estimation of interfacial interactions using ANN [38]. They reported the robust potential of the radial basis function for the prediction of interfacial interactions. Additionally, Tyagi et al. coupled artificial neural networks with genetic algorithms to optimize and model neodymium ion separation by a liquid membrane [39]. Furthermore, Rezakazemi et al. employed different ML approaches including ANFIS, PSO, and GA to estimate the selectivity of hydrogen in mixed matrix membranes [36]. Additionally, the maps for process evaluation for membrane-based CO_2_ separation technology were also developed by Gasós et al. using artificial neural networks [40].

In the current study, a robust intelligent model has been proposed to estimate the selectivity of mixed matrix membranes containing SAPO-34 zeolite for the separation of CO_2_ and CH_4_. The suggested model is novel, precise, and could anticipate the effect of different variables on the carbon dioxide and methane separation performance of MMMs. The proposed model also provides a grounding for synthesizing an MMM with the maximum carbon dioxide and methane separation efficiency.

## 2. Materials and Methods

### 2.1. Data Collection

As already discussed, SAPO-34 zeolite is one of the favorable zeolites in fabricated MMMs, which has been extensively investigated in the literature by several research groups [41,42,43,44,45,46,47,48,49,50,51,52]. On these grounds, an extensive experimental database of MMMs with different percentages of SAPO-34 zeolite was gathered from the literature [41,42,43,44,45,46,47,48,49,50,51,52] to ensemble a CNN-based paradigm for estimating the CO_2_/CH_4_ selectivity at different pressures and temperatures. Several substrates, including polyurethane, polysulfone, polytherysulfone, polyetherimide, Pebax 1657, Pebax 1074, and Matrimid 5218 have been checked in the literature [41,42,43,44,45,46,47,48,49,50,51,52]. As Table 1 shows, the database covers the filler dosage of 0–50 wt%, a temperature range of 298–348 K, and a pressure range of 0.1–3.0 MPa. In these ranges of experiments, the CO_2_/CH_4_ selectivity of MMMs varies from 1.38–66.99.

It must be mentioned that, although it is possible to simulate the effect of all influential variables on the CO_2_/CH_4_ selectivity using the CNN model, the following matters convinced us to only consider the effect of SAPO-34 dose, polymer type, temperature, and pressure on the selectivity.

1. The model is better to develop based on its easy and always available variables
2. Some of the potentially influential variables, including the MMM synthesis method and selectivity measurement procedure, are not reported in some of the original articles. Therefore, we have not considered them as independent variables.
3. It is better to ignore those variables that have a minor impact on the selectivity.

### 2.2. Dependency of CO_2_/CH_4_ Selectivity on Involved Variables

The previous section clearly defined both dependent and independent variables involved in methane and carbon dioxide separation by polymer/SAPO-34 membranes. Presently, Pearson’s method is applied to reveal the relationship between each pair of dependent and independent variables. This method uses Equation (1) to identify the most possible pattern between a pair of variables (i.e., *x* and *y*) and extract their relationship [53].

(1) $$PC_{x,y}\;=\;\sum_{k=1}^{m}\left(x_{k}-\bar{x}\right)\left(y_{k}-\bar{y}\right)/\left(\sqrt{\sum_{k=1}^{m}{\left(x_{k}-\bar{x}\right)}^{2}}\sqrt{\sum_{k=1}^{m}{\left(y_{k}-\bar{y}\right)}^{2}}\right)$$

Here $PC_{x,y}$ shows the Pearson’s coefficients between independent (*x*) and dependent (*y*) variables. In addition, *m* stands for the number of available records, and *k* is an index determining the upper and lower bounds of the summation operator. The relationship type/strength is coded using a coefficient that varies between −1 and +1 [54]. The minimum and maximum coefficients show the strongest indirect and direct relationships, respectively. The physical meaning of other coefficients can also be inferred by their closeness to either the maximum or minimum bounds.

**Figure 1:**
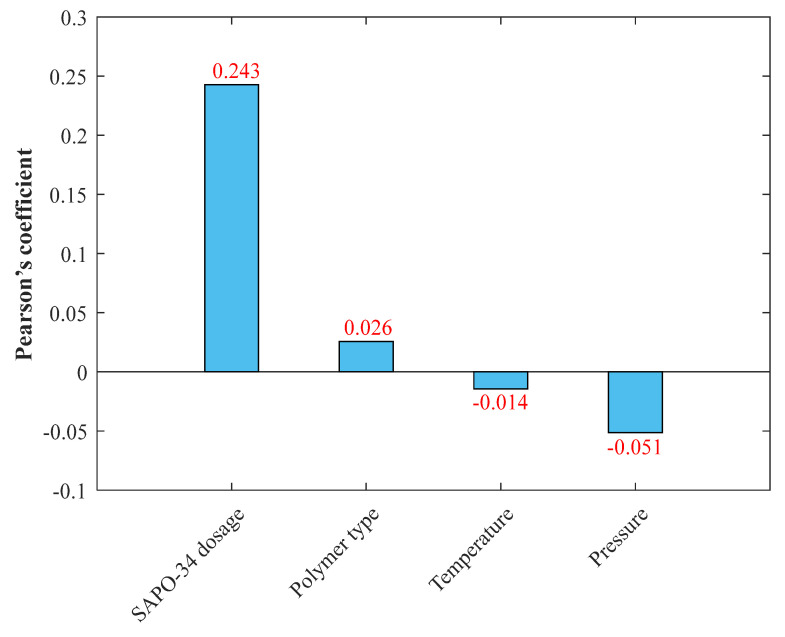
Pearson’s coefficients for the dependency of CO_2_/CH_4_ selectivity of MMMs to the involved features.

Figure 1 presents the Pearson’s coefficients for the relationship of CO_2_/CH_4_ selectivity to the SAPO-34 dosage in MMMs, polymer type, temperature, and pressure. The selectivity directly relates to the first two independent variables, while it indirectly relates to the two last variables. In addition, the selectivity dependency of the SAPO-34 dosage in MMMs and pressure are the strongest direct and indirect relationships, respectively.

It is better to note that the level of data scattering is a critical factor that often misleads the relevancy tests to provide inaccurate results. The literature clearly states that the Pearson’s predictions are sometimes the opposite of those approved by scientific facts [53]. Although Pearson’s method claimed that selectivity has almost no relationship with pressure and temperature, all experts in the field of membrane-based separation know that these are important variables and have a substantial impact on the operation.

It should also be mentioned that, since the polymer type is a qualitative variable, numerical indicators are applied to quantitatively present it. Indeed, polyurethane, polysulfone, polytherysulfone, polyetherimide, Pebax 1657, Pebax 1074, and Matrimid 5218 are coded by 1, 2, …, and 7, respectively.

### 2.3. Cascade Neural Networks (CNN)

ANN is a non-linear topology that was first developed according to the pattern of human brain processing for data analysis [55]. Accordingly, the flexibility, robustness, and accuracy of this strategy quickly nominated it in a broad range of applications from biomedicine to sustainable development [56,57]. It is possible to design a powerful ANN model to extract a logical pattern among the considered determinative factors, with related dependent ones, despite any degree of complexity [58]. To this end, providing an acceptable experimental dataset is a primary step in developing a black box for the prediction of targets [59]. In this way, the architecture of ANNs is obtained with signal analysis among the input and output factors. It is worth noting that the successful development of the ANN approach demands order specifications in different layers related to neuron interactions. Accordingly, various types of ANN topologies have been introduced such as multi-layer perceptron, cascade, radial basis function, and general regression [55]. Among these paradigms, the cascade neural network (CNN) is considered the most popular tool to simulate different phenomena [60,61]. The CNN approach directly connects the input layer to all the next hidden/output layers [60]. Accordingly, in the current study, the CNN topology has been employed which includes three key layers such as input, hidden, and output. Here, the input layer is associated with independent factors (variables) that have already affirmed the most significant traces of the process, which, after some data analyses, are sent to the hidden layer. In this layer, the major data processing and mathematical treatment are applied; thereafter, the outcomes are introduced to the output layer for final analysis. The applied mathematical processing related to neurons is specified by Equation (2) as follows [62]:

(2) $$NON=\sum_{r=1}^{M}w_{r}x_{r}+b$$

This equation states that the net output of a neuron (*NON*) can be calculated from the entry signal (i.e., $\left[x_{1},\;x_{1},\;\ldots ,\;x_{M}\right]{\;}^{T}$), weight vector (*ω_r_*), and a bias (*b*). Additionally, a transfer function (*f*) is supplied to receive the *NON* and calculate the outlet signal, i.e., *f(NON)*. Although there are various types of transfer functions [57], the current study uses the hyperbolic tangent sigmoid (Equation (3)) and logarithmic sigmoid (Equation (4)) in hidden and output layers, respectively [63].

(3) $$f\left(NON\right)=\frac{exp\left(NON\right)-exp\left(-NON\right)}{exp\left(NON\right)+exp\left(-NON\right)}$$

(4) $$f\left(NON\right)=\frac{1}{1+exp\left(-NON\right)}$$

It should be mentioned that to properly benefit from the normalization process to enhance the training rate as well as improve its quality, the logarithm sigmoid has been incorporated into the output layer. Indeed, all variables in our study have been normalized [0 1] before starting the model development phase.

### 2.4. Accuracy Measurement

This study relies on several accuracy criteria to measure the deviation between actual and predicted carbon dioxide to methane selectivity (*CMS*) of MMMs. The correlation of determination (*R*), absolute average relative deviation percent (*AARD%*), mean squared errors (*MSE*), relative deviation percent (*RD%*), and residual error (*RE*) are defined by Equations (5)–(9) and are used in the current study [64]:

(5) $$R=\sqrt{1-\;\left\{\sum_{k=1}^{m}{\left(CMS^{act}-CMS^{pred}\right)}_{k}^{2}/\sum_{k=1}^{m}{\left(CMS^{act}-\bar{CMS^{act}}\right)}_{k}^{2}\right\}}$$

(6) $$AARD\%=\;\left(100/m\right)\;\;\times \;{\sum_{k=1}^{m}\left(\left|CMS^{act}-CMS^{pred}\right|/CMS^{act}\right)}_{k}$$

(7) $$MSE=\;\left(1/m\right)\;\;\times \;{\sum_{k=1}^{m}\left(CMS^{act}-CMS^{pred}\right)}_{k}^{2}$$

(8) $$RD\%=\;100\;\times \;\left(CMS^{act}-CMS^{pred}\right)/CMS^{act}$$

(9) $$RE=\;\left(CMS^{act}-CMS^{pred}\right)$$

where *m* indicates the number of *CMS* samples. Furthermore, the *act* and *pred* superscripts are the actual and predicted values of the *CMS*.

It should be mentioned that the authors wrote several distinct codes in the Matlab environment and used each of them for a specific purpose: for (1) conducting the relevancy test, (2) constructing and testing the CNN model, (3) performing statistical analysis, and (4) creating graphs.

## 3. Results and Discussion

This section explains the process followed to determine the best topology of the CNN and evaluate its prediction accuracy.

### 3.1. Tuning the CNN Topology

Although it is possible to create the cascade neural network with arbitrary numbers of neuronic layers, this study estimates the CO_2_/CH_4_ selectivity of MMMs using a single hidden layer CNN. All the designed CNNs include an input layer, one hidden layer, and an output layer. Independent variables constitute the input layer and, therefore, it is fixed. In addition, the number of output neurons is dictated by the number of dependent variables (i.e., one). Therefore, it is only necessary to determine the best number of hidden neurons to fine-tune the CNN topology.

**Figure 2:**
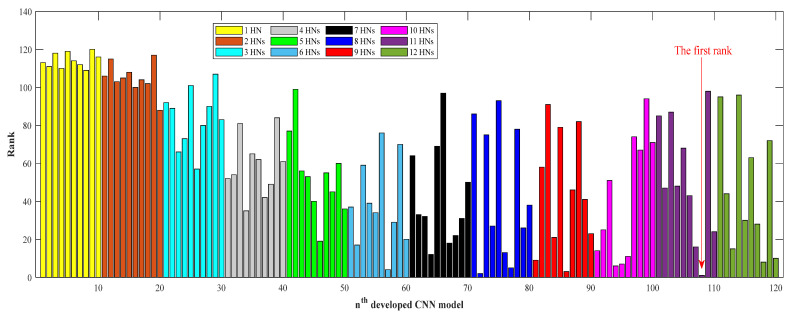
The procedure of the CNN topology tuning.

The numbers of the hidden neurons of CNNs are changed from one to twelve during a trial-and-error process. In addition, ten models are developed per each number of hidden neurons. In summary, this study develops 120 CNNs and compares their accuracy to find the highest accurate one. Figure 2 reports the results of the ranking analysis performed on the 120 developed CNNs. It can be seen that the eighth developed CNN model with eleven hidden neurons has the best performance and achieves the first-rank position. Therefore, the CNN model with eleven hidden neurons, a tangent sigmoid in the hidden layer, and a logarithm sigmoid in the output layer is selected as the final model to estimate the CO_2_/CH_4_ selectivity of MMMs.

Table 2 introduces the numerical values of the AARD%, MSE, and R indices related to the CNN performance to estimate 118 CO_2_/CH_4_ selectivity samples of polymer/SAPO-34 membranes. The training, testing, and all datasets have been estimated with excellent AARDs of 2.31%, 6.36%, and 2.93%, respectively. The structure-tuned CNN has also predicted these three datasets with the MSE of 0.51, 7.32, and 1.55, respectively. The closeness of the observed R values to the one in the training and testing steps, and their combination, is another indication of the outstanding ability of the proposed CNN for simulating the considered separation task.

**Figure 3:**
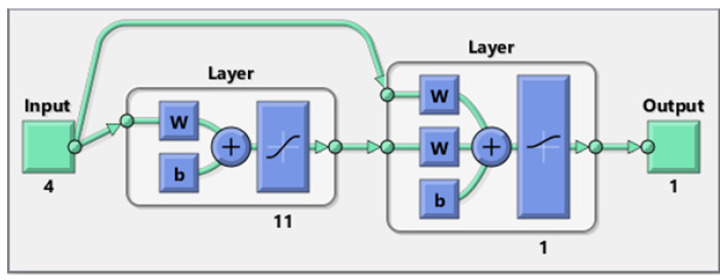
Schematic of the designed CNN for estimating the CO_2_/CH_4_ selectivity of MMMs [65].

The key information related to the designed CNN has been presented in Figure 3. The feedforward connection between input/hidden and hidden/output layers, as well as the cascade connection between input/output layers, are observable in this figure. Moreover, four inputs are SAPO-34 dosage, polymer type, pressure, and temperature, while the CO_2_/CH_4_ selectivity of MMMs is the only output. This figure also shows two neuronic layers (i.e., hidden and output) with 11 and 1 neurons, respectively. The tangent and logarithm sigmoid transfer functions can be easily seen in the hidden and output layers.

### 3.2. CNN Performance Evaluation

**Figure 4:**
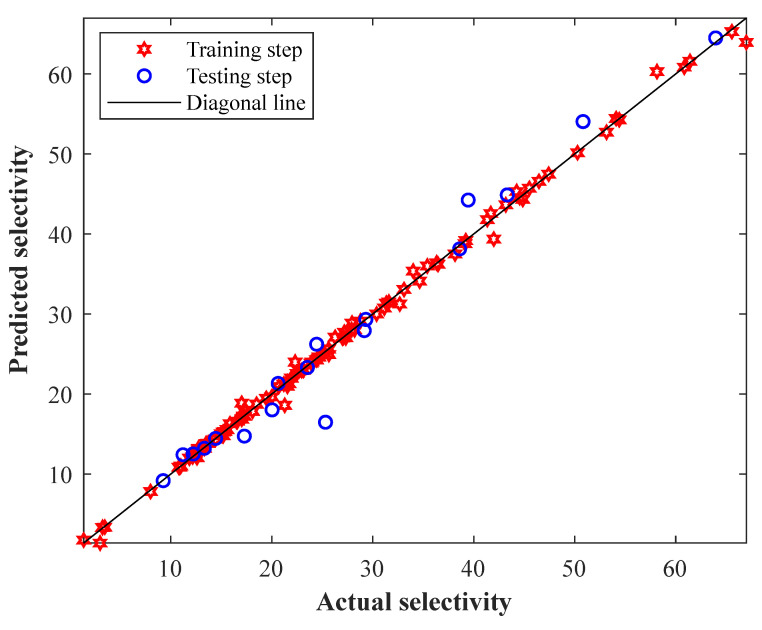
Cross-plot of the CNN predictions versus the actual CO_2_/CH_4_ selectivity of MMMs.

The cross-plot showing the predicted CO_2_/CH_4_ selectivity by the CNN versus actual values for the training, testing, and all datasets are depicted in Figure 4. This figure proves that the designed CNN has successfully mapped the estimated selectivity samples on their corresponding actual data in both training and testing steps. The R values related to the estimation of the training and testing groups are 0.9988 and 0.9860, respectively. These values state that the major parts of the predicted–actual symbols are located around the diagonal lines.

**Figure 5:**
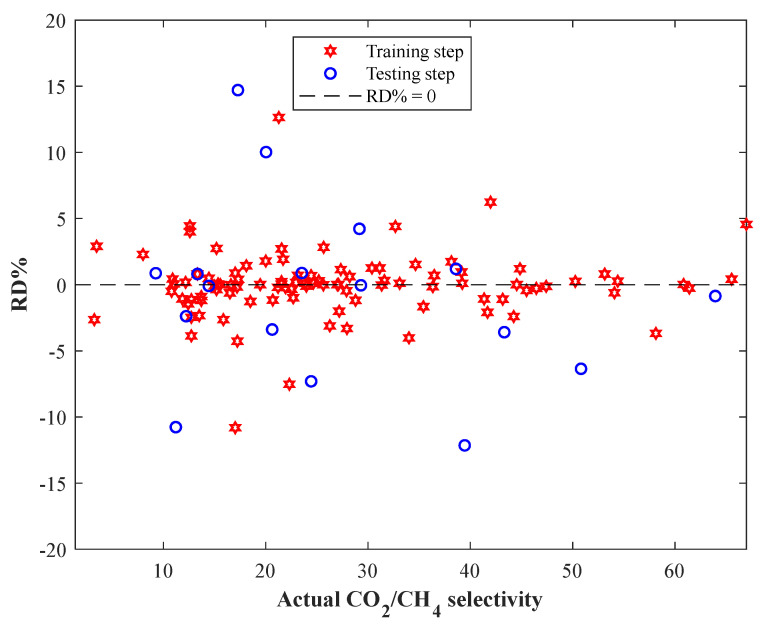
The observed RD% for estimating the CO_2_/CH_4_ selectivity of MMMs.

The relative deviation percent (i.e., Equation (8)) associated with estimating each experimental measurement of the actual CO_2_/CH_4_ selectivity of MMMs is shown in Figure 5. This figure shows that only four training and six testing CO_2_/CH_4_ selectivity samples have been estimated with an RD% higher than 5% or lower than −5%. It can be claimed that the proposed CNN model estimates 110 out of 118 actual CO_2_/CH_4_ selectivity samples with an excellent RD% in the range of −5% to 5%. 

**Figure 6:**
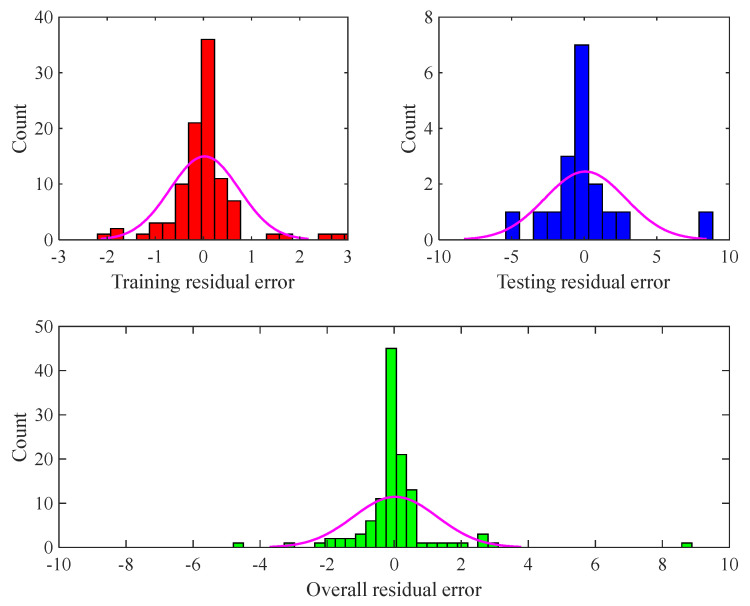
The observed residual error for estimating the training, testing, and overall databank.

The residual error between experimental and calculated CO_2_/CH_4_ selectivity samples (i.e., Equation (9)) for the training and testing phases, as well as all the datasets, has been illustrated in Figure 6. This figure shows that the CNN estimates almost all the experimental selectivity samples with the RE ranges from −3 to +3. Few CO_2_/CH_4_ selectivity samples are estimated with a RE outside this narrow range.

### 3.3. Validation by Experimental Measurements

**Figure 7:**
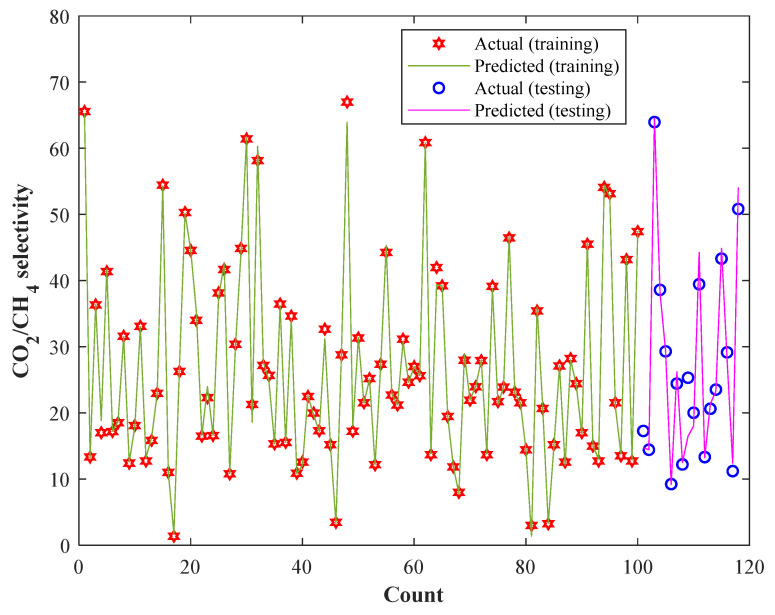
Actual CO_2_/CH_4_ selectivity of MMMs and their counterpart CNN estimations in the training as well as testing steps.

Figure 7 shows the experimental and CNN predictions for the training and testing sample of the CO_2_/CH_4_ selectivity of MMMs. Although the experimental CO_2_/CH_4_ selectivity measurements cover a relatively broad range (1.38–66.99) in different compositions of MMMs and operating conditions, the proposed CNN is able to precisely simulate the considered process. This figure also explains that 100 CO_2_/CH_4_ selectivity samples have been used in the training step and the remaining 18 samples are used in the testing step. It was previously reported that the proposed CNN estimates the training and testing datasets with excellent AARDs of 2.31% and 6.36%, respectively.

### 3.4. Investigating the Effect of Involved Features on the Selectivity

**Figure 8:**
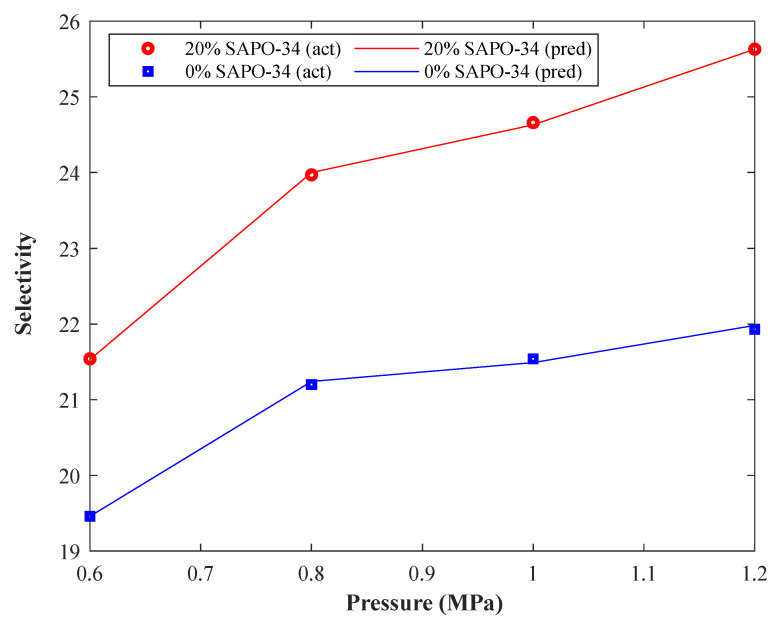
The dependency of CO_2_/CH_4_ selectivity of polyurethane-based MMMs on the pressure and filler dose (298 K).

Figure 8 introduces experimental records and CNN predictions for the CO_2_/CH_4_ selectivity of polyurethane membrane and polyurethane-SAPO-34 MMM versus pressure at 298 K. This figure justifies the acceptable agreement between actual and predicted selectivity samples. This figure also shows that the CO_2_/CH_4_ selectivity of both membranes increases with increasing pressure and filler dose. Although Pearson’s method correctly anticipated the filler effect on the selectivity, it provided a wrong result for the selectivity–pressure relationship. As mentioned before, this wrong prediction of the relevancy test is often related to the high level of scattering in the experimental data.

It is better to note that the relevancy test also anticipated a weak relationship between selectivity and pressure, but this analysis approves that the pressure is able to change selectivity sharply.

**Figure 9:**
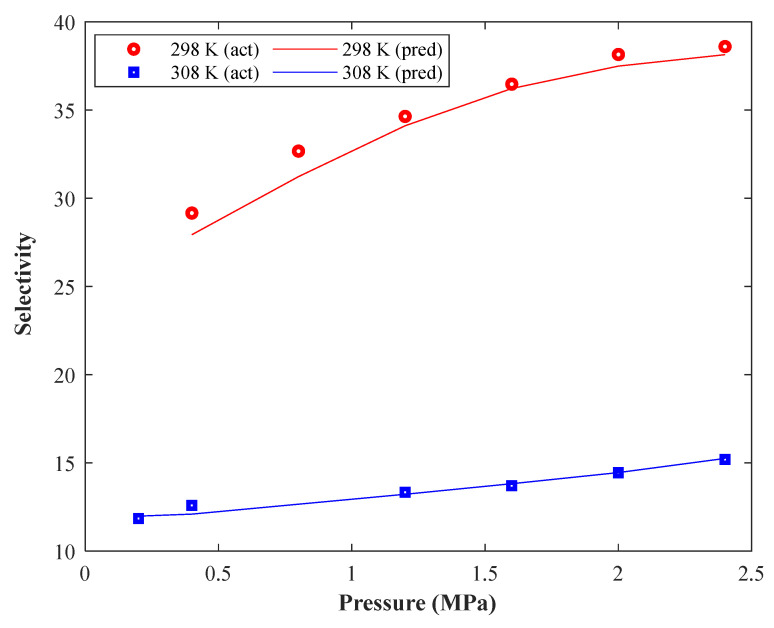
The dependency of CO_2_/CH_4_ selectivity of Pebax 1074-based MMMs on the pressure and temperature (20% SAPO-34).

The pressure effect on the CO_2_/CH_4_ selectivity of Pebax 1074-based MMMs containing 20% SAPO-34 at two temperature levels (298 and 308 K) is depicted in Figure 9. The increasing effect of pressure and decreasing effect of temperature on the CO_2_/CH_4_ selectivity can be concluded from this figure. The relevancy test also confirmed an indirect relationship between selectivity and temperature.

It is better to note that Pearson’s method also claimed that a weak relationship exists between selectivity and temperature, but this analysis approves that the temperature impact on the selectivity is strong.

## 4. Conclusions

This study aimed to estimate the CO_2_/CH_4_ selectivity of those mixed matrix membranes composed of polymeric substrates and with SAPO-34 zeolite as the filler. The cascade neural network was chosen to extract the relationship between the CO_2_/CH_4_ selectivity of the considered MMMs and the involved independent variables (i.e., filler dosage in polymeric matrices, polymer type, temperature, and pressure). Pearson’s method proved that an acceptable degree of relevancy existed between the selectivity and the influential variables. This method identified that the SAPO-34 dosage in the composite membrane has the strongest direct effect on the CO_2_/CH_4_ selectivity of the considered MMMs. The CNN topology has been fine-tuned using trial-and-error and statistical analyses. The CNN with 11 hidden nodes estimate 118 actual selectivity samples with the AARD = 2.92%, MSE = 1.55, and R = 0.9964, is identified as the most accurate model for the considered task. Moreover, several graphical analyses, including cross-plot, residual error, relative deviation percent, and predicted versus actual graphs have further justified the outstanding performance of the proposed CNN for estimating the CO_2_/CH_4_ selectivity of the MMMs. Such a reliable model helps monitor the effect of MMM composition (polymer type and filler dosage) and operating conditions (pressure and temperature) on the potential CO_2_/CH_4_ selectivity.

## Figures and Tables

**Table 1:** Summary of the reported CO_2_/CH_4_ selectivity of MMMs in the literature [41,42,43,44,45,46,47,48,49,50,51,52].

Variable	Observations	Minimum	Maximum	Mean	St. Deviation
Filler dosage (wt%)	118	0	50	12.11	11.11
Temperature (K)	118	298	348	305.03	10.51
Pressure (MPa)	118	0.10	3.0	0.93	0.67
CO_2_/CH_4_ selectivity	118	1.38	66.99	26.69	14.62

**Table 2:** Information on the obtained AARD%, MSE, and R by the best CNN model.

Dataset	AARD%	MSE	R
Training step	2.31	0.51	0.9988
Testing step	6.36	7.32	0.9860
All the data	2.93	1.55	0.9964

## Data Availability

The study data analyzed in this article can be obtained by request from the corresponding author.
